# Real‐world data for lenalidomide maintenance in responding patients of diffuse large B‐cell lymphoma

**DOI:** 10.1002/cam4.5790

**Published:** 2023-03-13

**Authors:** Xiaoyan Wang, Lu Yu, Xinlu Jiang, Kaiyang Ding

**Affiliations:** ^1^ Department of Hematology, Anhui Provincial Hospital Anhui Medical University Hefei China; ^2^ Department of Hematology, Anqing Municipal Hospital Anhui Medical University Anqing China; ^3^ Department of Hematology, the First Affiliated Hospital of USTC, Division of Life Sciences and Medicine University of Science and Technology of China Hefei China

**Keywords:** diffuse large B‐cell lymphoma, lenalidomide, maintenance therapy, R‐CHOP, recurrence

## Abstract

**Background:**

Approximately 40% patients of diffuse large B‐cell lymphoma (DLBCL) would develop disease recurrence/progression after first‐line R‐CHOP (rituximab, cyclophosphamide, doxorubicin, vincristine, and prednisone) induction therapy, with highly poor prognosis. An effective strategy to prolong the survival of this patient population is the additional single‐drug maintenance therapy. lenalidomide, an immunomodulatory drug with oral activity, has direct anti‐tumor activity and indirect effects mediated by multiple immune cells in the tumor microenvironment, such as B, T, natural killer (NK), and dendritic cells. Combining its controllable toxicity, it is promising in long‐term maintenance therapy. This study aims at evaluating the clinical effect of lenalidomide maintenance therapy in responding DLBCL patients with R‐CHOP treatment.

**Methods:**

This retrospective study was devised in DLBCL cases who obtained complete response (CR) or partial response (PR) following 6–8 cycles of R‐CHOP treatment between January 1, 2015 and July 31, 2019. Patients (*n* = 141) included were respectively assigned to receive lenalidomide maintenance treatment (lenalidomide, *n* = 50) and drug‐free maintenance treatment (control, *n* = 91) after CR/PR. lenalidomide was provided orally at 25 mg/day for 10 days, with a cycle of 21 days and a treatment course of 2 years. Progression‐free survival (PFS) was taken as the primary outcome.

**Results:**

Of the total 141 subjects, the median follow‐up time was 30.9 months (range, 5.7–68.9 months). The 2‐year PFS was 84% (95% CI: 74%–94%) in the lenalidomide group and 53% (95% CI: 43%–63%) in the control group. The median PFS of the lenalidomide group was not reached, and that of the control group was 42.9 months (HR = 0.32; 95% CI: 0.16–0.63; *p* = 0.001). No remarkable difference in overall survival (OS) between the two groups was indicated (HR = 0.42; 95% CI: 0.16–1.12; *p* = 0.08). Central nervous system (CNS) recurrence happened in 5 patients (5.5%) of the control group, while none of the patients with lenalidomide had CNS recurrence. Additionally, neutropenia and cutaneous reactions were the most common Grade 1–2 adverse reactions after lenalidomide treatment, and neutropenia was the most frequent Grade 3–4 adverse reaction.

**Conclusion:**

Two‐year lenalidomide maintenance treatment can significantly prolong the PFS of DLBCL patients who obtained CR/PR to first‐line R‐CHOP treatment.

## Graphical abstract

We retrospectively analyzed 141 DLBCL patients who responded to R‐CHOP and the patients included were respectively assigned to receive Lenalidomide maintenance treatment (Lenalidomide, n = 50) and drug‐free maintenance treatment (Control, n = 91). We found that two‐year Lenalidomide maintenance treatment can significantly prolong the PFS of DLBCL patients who obtained CR/PR to first‐line R‐CHOP treatment, and reduce the incidence of CNS recurrence.

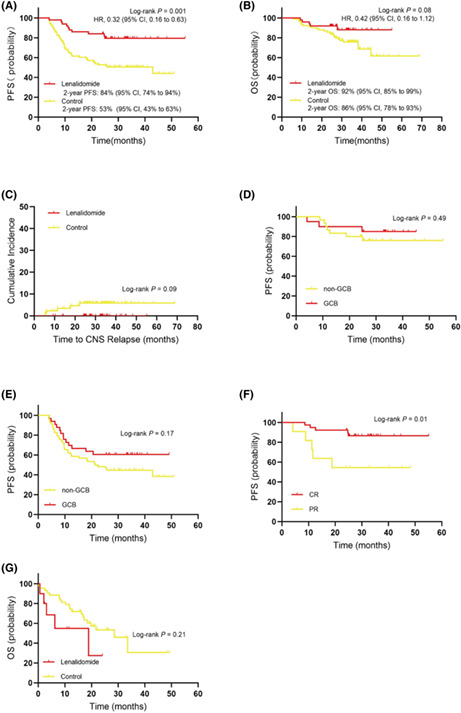

## 1 INTRODUCTION

Diffuse large B‐cell lymphoma (DLBCL), the most prevalent non‐Hodgkin's lymphoma (NHL) in adults, is predicted to account for 32.5% in newly diagnosed NHL cases annually and 40% in total lymphoma cases in the world.^1^, ^2^ In China, the burden of lymphoma has been rising in the past decade.^3^ Currently, the R‐CHOP regimen (rituximab, cyclophosphamide, doxorubicin, vincristine, and prednisone) is regarded as the standard first‐line therapy in newly diagnosed DLBCL patients of all ages.^4^ In that way, patient response combined with [^18^F] 2‐fluoro‐2‐deoxy‐D‐glucose positron emission tomography/computed tomography (FDG‐PET/CT) assessment appears to be important in decision‐making of subsequent treatment strategy.^4^ DLBCL is curable while there is still a risk (>30%) of recurrence after first‐line treatment leading to highly poor prognosis. As reported, patients who developed disease relapse after treatment had only 41% and 27% survival rate in 1 year and 5 years, respectively, with the median survival time of only 10 months.^5^ According to the Chinese Guidelines for the Diagnosis and Treatment of diffuse Large B‐cell Lymphoma (2013 Version), second‐line chemotherapy with non‐cross‐resistance to CHOP is recommended for DLBCL patients after relapse, such as rituximab plus ifosfamide, carboplatin, and etoposide (R‐ICE), rituximab plus dexamethasone, cytarabine, and cisplatin (R‐DHAP), rituximab plus etoposide, methylprednisolone, cytarabine, and cisplatin (R‐ESHAP), rituximab plus gemcitabine, dexamethasone, and cisplatin (R‐GDP), rituximab plus gemcitabine and oxaliplatin (R‐GemOx), rituximab plus dose‐adjusted etoposide, prednisone, vincristine, cyclophosphamide, and doxorubicin (R‐DAEPOCH).^6^ Autologous stem cell transplantation (ASCT) will be performed after salvage therapy if the patient achieves complete or partial remission and has the conditions for transplantation. If the patient does not have the conditions for transplantation or the disease status remains stable or progressive after treatment, the clinical trial will be recommended. Although ASCT is the standard treatment for relapsed DLBCL patients, half of the patients are ineligible for transplantation due to failure of salvage therapy, and the other half will relapse after ASCT.^7^ According to the study of CORAL, 3‐year PFS was only 21% in patients who had previously treated with rituximab and receiving R‐ICE or R‐DHAP prior to ASCT.^8^ The poor prognosis, drug toxicity caused by salvage therapy, increasing hospitalization times, and the loss of labor force have brought great psychological and economic burden to relapsed DLBCL patients. Therefore, it is particularly important to improve the complete remission rate and maintain the remission state in first‐line treatment. Many attempts were made to increase the efficacy of R‐CHOP by applying cytotoxic drugs or increasing the dose administrated, yet there was no improvement in outcomes except in specific populations.^9^, ^10^, ^11^, ^12^, ^13^, ^14^, ^15^, ^16^ Under this circumstance, single‐drug maintenance therapy has been tried after initial treatment with R‐CHOP. It is expected to improve the disease control after initial therapy and eliminate residual disease, thereby to delay disease progression, increase long‐term survival and ultimately get disease cured. In most cases, relapse occurs 12–18 months after initial therapy. There was a study reporting that DLBCL patients who survived uneventfully in 2 years had an overall survival (OS) rate consistent with normal individuals, highlighting the significance of maintaining disease condition within this time period.^17^ Many drugs have been applied in maintenance therapy in patients with DLBCL, such as rituximab, enzastaurin, everolimus, and lenalidomide, while only lenalidomide, a type of orally active immunomodulatory drug, has shown significant effects on PFS.^18^, ^19^, ^20^, ^21^ Rituximab is not recommended in maintenance therapy in DLBCL with first‐line R‐CHOP as it is a part of the treatment.^4^ Lenalidomide, different with conventional chemotherapy and rituximab in mechanism of action, has direct anti‐tumor activity and immunomodulatory effects.^22^, ^23^, ^24^ It has shown significant activity and is safe in patients having recurrent DLBCL and in combination with R‐CHOP, which provides potent theoretical evidence for lenalidomide maintenance in responding DLBCL cohort receiving R‐CHOP therapy.^25^, ^26^, ^27^, ^28^, ^29^ This study retrospectively analyzed the role of lenalidomide in DLBCL cases who obtained CR or PR to R‐CHOP initial treatment.

## 2 METHODS

### 2.1 Study cohort

This retrospective study was devised in DLBCL cases who obtained PR/CR to R‐CHOP therapy between January 1, 2015 and July 31, 2019 in Anhui Provincial Cancer Hospital were included. Patients were assessed as CR/PR after 4 cycles of R‐CHOP. Patients with the characteristics will be considered for lenalidomide maintenance therapy, such as the medium‐high‐risk group/high‐risk group according to NCCNIPI; Ann Arbor stage III/IV or extranodal lesions (bone marrow, CNS, liver, gastrointestinal tract, lung, and other important organs); Double expression or mutation of *MYC*, *BCL2*, or *BCL6* genes. Written informed consent was obtained prior to the treatment from all subjects. Eligible patients were ≥ 18 years old with histologically confirmed DLBCL, other inclusion criteria included completion of 6–8 cycles of R‐CHOP and CR or PR was assessed at the end of induction therapy; maintenance therapy with lenalidomide or maintenance therapy without any drugs; clinical data were complete, with measurable or evaluable indicators. Patients with incomplete clinical data, spontaneous interruption of maintenance therapy with lenalidomide, or replacement of maintenance drugs were excluded from this study. The median interval time from the end of induction therapy to the beginning of oral lenalidomide was 4 months. Of the 150 people initially included (lenalidomide: *n* = 50; control: *n* = 100), 9 people in the control group had disease progression within 4 months after induction therapy and thus excluded. Eventually, 141 subjects were recruited (lenalidomide: *n* = 50; control: *n* = 91).

### 2.2 Treatment schedule and therapeutic outcomes

In the lenalidomide group, lenalidomide was given with an initial dose of 25 mg/day (10 mg/day for patients with 30–60 mL/min creatinine clearance [CrCI]) and gradually adjusted according to patient tolerance, from day 1 to day 10, 21 days as a cycle (we used 10/21 days as per our past experience of toxicity). Lenalidomide was maintained when disease progressed or an unacceptable event occurred. Treatment response was evaluated according to the Lugano classification.^30^ CT and/or FDG‐PET/CT scans were performed to assess treatment response, and MRI was performed to evaluate the CNS. PET‐CT scans will be performed at 2–4 cycles for evaluation after initiation of lenalidomide. The primary outcome was PFS, a time period between the beginning of the study and the occurrence of disease progression or disease‐related death. The secondary outcomes were OS, 2‐year PFS, CNS recurrence, and safety. Medical record review and telephone interview were conducted to assess the incidence of treatment‐related hematological and non‐hematological adverse events.

### 2.3 Data analysis

In the data analysis, the chi‐squared test and Fisher's exact test were used for categorical variables, and the Mann–Whitney rank‐sum test was used for continuous variables to compare the subject characteristics. PFS and OS were estimated by the Kaplan–Meier method with the log‐rank test applied in between‐group comparisons, and the GraphPad Prism 8 was run to plot survival curves. Cox regression models were established to analyze factors prognostic for disease progression, and the variables on a significance level of 0.10 in the univariate model were selected and further analyzed in a multivariate model. Significance of a difference was defined by *p* ≤ 0.05. SPSS 25 software was operated to complete all data analyses.

## 3 RESULTS

### 3.1 Patient characteristics

In all, 141 patients having a definite diagnosis of DLBCL and receiving R‐CHOP therapy were analyzed (lenalidomide: *n* = 50, control: *n* = 91). The median age at diagnosis was 55 years in both groups. CR was achieved in 104 patients (lenalidomide: *n* = 39, control: *n* = 65) and PR was achieved in 37 patients (lenalidomide: *n* = 11, control: *n* = 26). In both groups, germinal center B‐cell‐like (GCB) DLBCL was less frequent versus non‐GCB DLBCL (lenalidomide: 40% vs. 60%, control: 36.3% vs. 63.7%). Advanced disease was seen in 62% of the lenalidomide group and 62.6% of the control group (*p* = 0.94). Bone marrow involvement occurred in 14% and 8.8% in the lenalidomide and control groups respectively (*p* = 0.34). CNS IPI of 4–6 was seen in 20% of the lenalidomide group and 31.9% of the control group (*p* = 0.17). Myc immunohistochemistry information was available for 116 patients (116/141, 82.3%). A total of 34 patients (34/116, 29.3%) were double expressor at baseline. The patient demographic data and baseline disease characteristics at diagnosis were detailed in Table 1.

**TABLE 1:** Demographic patient characteristics at diagnosis.

Characteristic	Lenalidomide (*n* = 50)	Control (*n* = 91)	*p*
Age, years			
Median (range)	55 (19–83)	55 (21–84)	0.69
< 60	32 (64.0)	49 (53.8)	0.24
Sex			
Male	24 (48.0)	54 (59.3)	0.20
Female	26 (52.0)	37 (40.7)	
GCB/non‐GCB profile (by Hans algorithm)			
GCB	20 (40.0)	33 (36.3)	0.66
Non‐GCB	30 (60.0)	58 (63.7)	
ECOG performance status			
< 2	39 (78.0)	67 (73.6)	0.57
≥ 2	11 (22.0)	24 (26.4)	
Ann Arbor clinical stage			
I–II	19 (38.0)	34 (37.4)	0.94
III–IV	31 (62.0)	57 (62.6)	
NCCN‐IPI			
0–3	30 (60.0)	48 (52.7)	0.41
4–8	20 (40.0)	43 (47.3)	
No. of extranodal sites			
≤ 1	35 (70.0)	70 (76.9)	0.37
> 1	15 (30.0)	21 (23.1)	
B symptoms			
Yes	18 (36.0)	32 (35.2)	0.92
No	32 (64.0)	59 (64.8)	
Bulky mass (>10 cm)			
Yes	11 (22.0)	21 (23.1)	0.88
No	39 (78.0)	70 (76.9)	
Response to R‐CHOP			
CR	39 (78.0)	65 (71.4)	0.40
PR	11 (22.0)	26 (28.6)	
Elevated LDH (>ULN)			
Yes	23 (46.0)	44 (48.4)	0.79
No	27 (54.0)	47 (51.6)	
β2 microglobulin			
< 2.8 mg/L	27 (54.0)	49 (53.8)	0.99
> 2.8 mg/L	23 (46.0)	42 (46.2)	
Bone marrow involvement			
Yes	7 (14.0)	8 (8.8)	0.34
No	43 (86.0)	83 (91.2)	
CNS‐IPI			
0–1	10 (20.0)	22 (24.2)	0.17
2–3	30 (60.0)	40 (44.0)	
4–6	10 (20.0)	29 (31.9)	
Testicular involvement			
Yes	2 (4.0)	4 (4.4)	1.00
No	48 (96.0)	87 (95.6)	
Double expression			
Yes	8 (16.0)	26 (28.6)	0.09
No	33 (66.0)	49 (53.8)	
Missing	9 (18.0)	16 (17.6)	

*Note*: Data are presented as No. (%) unless otherwise noted.

Abbreviations: CR, complete response; ECOG, Eastern Cooperative Oncology Group; GCB, germinal center B‐cell like; LDH, lactate dehydrogenase; NCCN‐IPI, National Comprehensive Cancer Network International Prognostic Index; PR, partial response; R‐CHOP, rituximab, cyclophosphamide, doxorubicin, vincristine, and prednisone; ULN, upper limit of normal.

### 3.2 PFS

**FIGURE 1:**
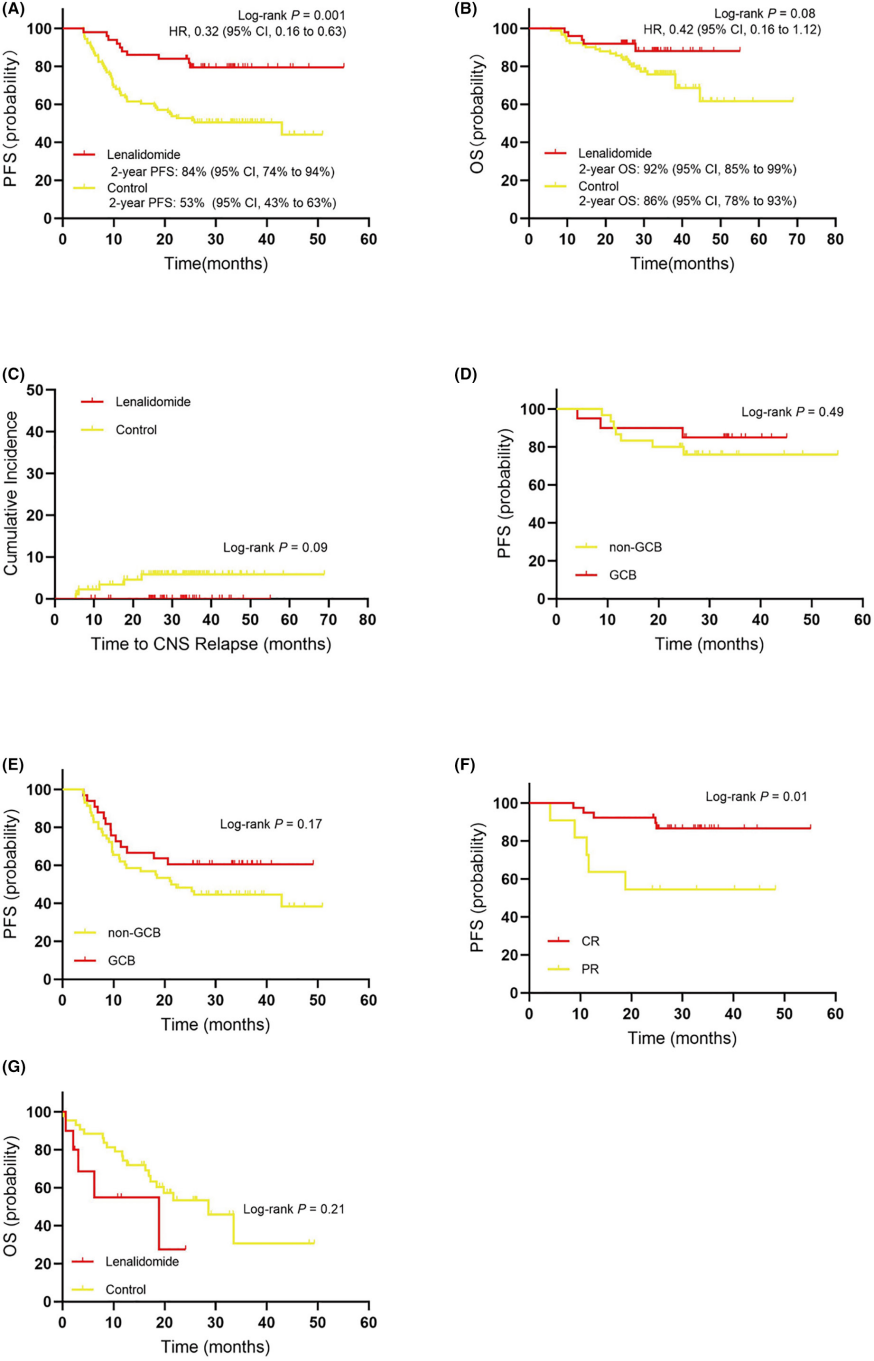
(A) Progression‐free survival (PFS) in all patients. (B) Overall survival (OS) in all patients. (C) The lenalidomide group shows a tendency of better cumulative risk of CNS relapse compared to the control group although their difference is not statistically significant. (D) The PFS is not different between patients with germinal center B‐cell‐like (GCB) type and patients with non‐GCB type in the lenalidomide group. (E) The PFS is not different between patients with GCB type and patients with non‐GCB type in the control group. (F) The PFS of patients who have achieved complete response (CR) is better than those who have achieved partial response (PR). (G) OS after disease progression in the lenalidomide and control groups.

**FIGURE 2:**
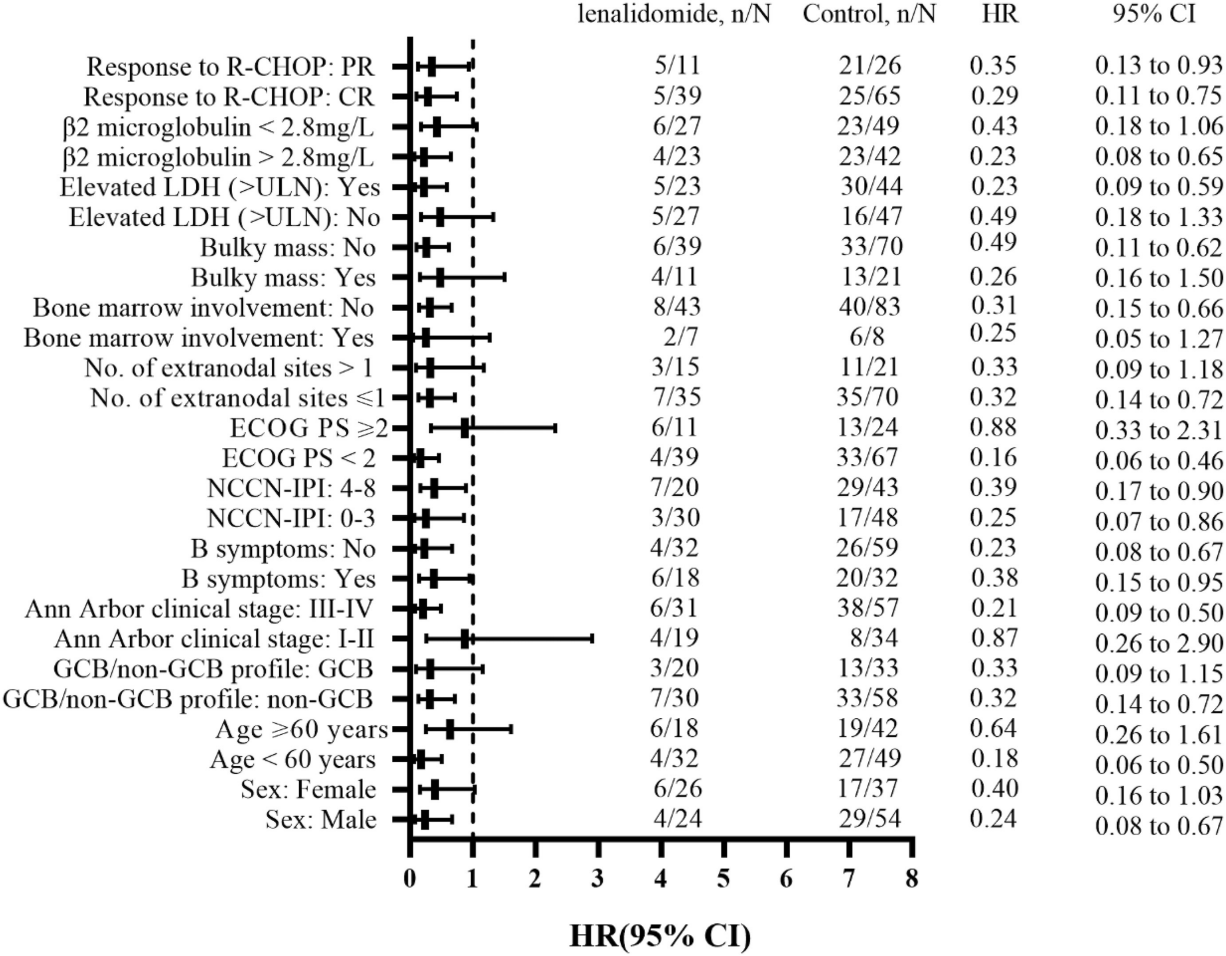
Subgroup analysis of progression‐free survival (PFS). HR <1 favors lenalidomide group, HR >1 favors the control group. Abbreviations: CR, complete response; ECOG PS, Eastern Cooperative Oncology Group Performance Status; GCB, germinal center B‐cell like; LDH, lactate dehydrogenase; NCCN‐IPI, National Comprehensive Cancer Network International Prognostic Index; PR, partial response; R‐CHOP, rituximab, cyclophosphamide, doxorubicin, vincristine, and prednisone; ULN, upper limit of normal.

The median follow‐up time was 30.9 months (range, 5.7–68.9 months). The median PFS of patients with lenalidomide was not reached and that of control was 42.9 months (HR = 0.32; 95% CI: 0.16–0.63; *p* = 0.001) (Figure 1A). The 2‐year PFS was 84% (95% CI: 0.74–0.94) in the lenalidomide cohort and 53% (95% CI: 0.43–0.63) in the control cohort. The PFS benefits were significant in all subgroups with lenalidomide maintenance treatment (Figure 2), and the PFS of patients who achieved CR at initial R‐CHOP therapy was better than that of patients achieving PR (Figure 1F). Moreover, among the 11 PR patients, 3 PR patients got converted into a CR during lenalidomide maintenance. 42 patients have completed full 2 years of lenalidomide maintenance. Eight patients progressed during maintenance, of which six patients progressed in the first year and two patients progressed in the second year.

### 3.3 OS

The median OS of both groups were not reached, and the between‐group difference was not remarkable (HR = 0.42; 95% CI: 0.16–1.12; *p* = 0.08) (Figure 1B). The 2‐year OS was 92% (95% CI: 0.85–0.99) in the lenalidomide group and 86% (95% CI: 0.78–0.93) in the control group.

### 3.4 Secondary CNS involvement

CNS recurrence occurred in 5 (5.5%) control patients versus 0 (0%) with lenalidomide (*p* = 0.09) (Figure 1C). Of the five patients, three cases had >1 extranodal involvement, four had bone involvement, four had Stage IV disease, three had non‐GCB DLBCL, one had CNS IPI of 0–1, two had CNS IPI of 2–3, and two had CNS IPI of 4–6. None of the patients received treatment preventive for CNS recurrence. The time to develop CNS recurrence from the end of R‐CHOP in these five patients was 22.2, 11.4, 6.1, 5.4, and 17.7 months, respectively. The 2‐year CNS recurrence rate estimated by the Kaplan–Meier method was 6% in the control and 0 in the lenalidomide cohort.

### 3.5 Cell of origin

According to the HANS classification criteria, DLBCL subtypes include GCB and non‐GCB. Of the non‐GCB cases, the median PFS between the lenalidomide group (not reached) and the control group (21.3 months) was statistically significant (*p* = 0.004). No remarkable difference was indicated in the median PFS of GCB patients between the two cohorts (*p* = 0.07). Regarding OS, no evident difference was noted in GCB (*p* = 0.90) versus non‐GCB (*p* = 0.07) cohorts of the two groups. Additionally, the PFS in GCB and non‐GCB cases was marginally different in both groups (lenalidomide: *p* = 0.49, control: *p* = 0.17) (Figure 1D; Figure 1E). Regardless of disease type, patients in the lenalidomide group had better survival outcome versus the patients in the control group.

### 3.6 Prognostic factors

Univariate analysis was performed and revealed the group, age, ECOG performance status, Ann Arbor clinical stage, NCCN‐IPI, B symptoms, response to R‐CHOP, LDH level, bone marrow involvement, bulky mass (>10 cm), and GCB/non‐GCB profile (by Hans algorithm) on a significance level of 0.01 (Table 2). With the inclusion of these significant factors (including age of clinical significance), COX multivariate regression analysis was subsequently devised. The group (lenalidomide; HR = 0.30; 95% CI: 0.15–0.60; *p* = 0.001), NCCN‐ IPI (4–8; HR, 2.67; 95% CI: 1.06–6.74; *p* = 0.04), and response to R‐CHOP (PR; HR = 3.29; 95% CI: 1.86–5.81; *p* = 0.00) were independently prognostic for PFS. GCB/non‐GCB profile (GCB; HR = 0.31; 95% CI: 0.12–0.82; *p* = 0.02), NCCN‐IPI (4–8; HR = 29.84; 95% CI: 4.67–190.74; *p* = 0.00), B symptoms (HR = 2.60; 95% CI: 1.10–6.12; *p* = 0.03), response to R‐CHOP (PR; HR = 2.73; 95% CI: 1.20–6.24; *p* = 0.02), and LDH level (elevated; HR = 0.28; 95% CI: 0.10–0.80; *p* = 0.02) were independently prognostic for OS.

**TABLE 2:** Univariate prognostic factors.

Characteristic	PFS	OS
	*p*	*p*
Group	0.00	0.08
Age, years	0.58	0.03
Sex	0.68	0.91
GCB/non‐GCB profile	0.11	0.06
ECOG performance status	0.02	0.01
Ann Arbor clinical stage	0.00	0.01
NCCN‐IPI	0.00	0.00
No. of extranodal sites	0.97	0.98
B symptoms	0.03	0.01
Bulky mass (> 10 cm)	0.04	0.02
Response to R‐CHOP	0.00	0.00
Elevated LDH (> ULN)	0.00	0.05
β2 microglobulin	0.50	0.16
Bone marrow involvement	0.14	0.09

Abbreviations: ECOG, Eastern Cooperative Oncology Group; GCB, germinal center B‐cell like; LDH, lactate dehydrogenase; NCCN‐IPI, National Comprehensive Cancer Network International Prognostic Index; R‐CHOP, rituximab, cyclophosphamide, doxorubicin, vincristine, and prednisone; ULN, upper limit of normal.

### 3.7 Safety

During the treatment with lenalidomide, neutropenia of varying degrees occurred in 31 patients (31/50, 62%), and Grade 3–4 neutropenia occurred in 14 patients (14/50, 28%). Infection was seen in 7/50 (14%) cases, including 4 concurrently with Grade 3–4 neutropenia and 2 with Grade 3–4 infections (1 with neutropenia). Anemia developed in 9/50 (18%) patients. Thrombocytopenia happened in 14/50 (28%) patients, including Grade 3–4 in 2 patients. Grade 3–4 cutaneous reactions occurred in 1/50 (2%) patient. Acute myocardial infarction requiring emergency treatment was seen in 1/50 (2%) patient. Due to hematologic toxicity and severe cutaneous reactions, 5/50 (10%) patients received lenalidomide with a dose reduction and completed 2 years of maintenance therapy. No patient discontinued lenalidomide due to grade 3–4 adverse events. There was no treatment‐related death (Table 3).

**TABLE 3:** Feasibility and toxicity of lenalidomide maintenance.

	Grade 1–2	Grade 3–4	Grade 5
Neutropenia	17 (34)	14 (28)	0
Anemia	9 (18)	0	0
Thrombocytopenia	12 (24)	2 (4)	0
Infection	5 (10)	2 (4)	0
Hepatotoxicity	2 (4)	0	0
Cardiac disorders	0	1 (2)	0
Cutaneous reaction	13 (26)	1 (2)	0
Fatigue	4 (8)	0	0

*Note*: Data are presented as No. (%) unless otherwise noted.

## 4 DISCUSSION

In this study, a comparative analysis was devised with the real‐world clinical data, to discuss the efficacy of lenalidomide maintenance in DLBCL patients having CR/PR to R‐CHOP therapy. As analyzed, lenalidomide maintenance therapy exhibited superior effect on PFS (lenalidomide vs. control; HR = 0.32; 95% CI: 0.16–0.63; *p* = 0.001), while only showed minor OS benefit (lenalidomide vs. control; HR = 0.42; 95% CI: 0.16–1.12; *p* = 0.08). This is similar to the finding of a previous phase III study,^21^ where the patients with placebo treatment received successful salvage therapy after disease progression, which was considered a cause of the similar OS outcome between the lenalidomide maintenance treatment and the placebo treatment by some researchers.^31^ In the present study, median OS after disease progression was compared between the two groups, and found to be 18.9 months (95% CI: 2.9–34.8 months) in the lenalidomide group and 21.7 months (95% CI: 10.0–33.5 months) in the control group (*p* = 0.21) (Figure 1G), remarkably longer versus the median OS at 10 months after recurrence in the previous study.^5^ This could be attributable to the salvage therapy with an addition of some new drugs, thereby evidently improving the prognosis of patients. Of note, it remains to be validated the cause of minor OS benefit by lenalidomide maintenance therapy, requiring further long‐term follow‐up and analysis in a larger population.

Lenalidomide is an immunomodulatory drug of oral activity. It has direct anti‐tumor activity and also exhibits indirect effects which are mediated by a variety of immune cells, including B, T, natural killer (NK), and dendritic cells, located to the tumor microenvironment.^22^ It was reported that lenalidomide was effective in the salvage therapy and the subsequent maintenance therapy in relapsed/refractory primary central nervous system lymphoma (PCNSL), as it could penetrate the ventricle and get into the cerebrospinal fluid.^32^, ^33^, ^34^ In the REMARC study, 2 years of lenalidomide maintenance therapy was independent of lower CNS recurrence.^35^ To the contrary, in a study reporting DLBCL with R‐CHOP therapy, addition of lenalidomide contributed to a lower rate of CNS recurrence.^36^ In the present study, the CNS recurrence rate was 5/91 (5.5%) in the control group, while none of the patients with Lenalidomide maintenance therapy had CNS recurrence (*p* = 0.09). Moreover, CNS IPI of 4–6 was seen in 20% (10/50) of the lenalidomide group and 31.9% (29/91) of the control group (*p* = 0.17). More patients in control group had high CNS IPI. It is difficult to figure out Len's contribution for CNS protection as more patients had high CNS IPI in the control group. Such phenomenon prompts us to devise larger prospective studies on the role of lenalidomide in decreasing CNS recurrence and the timing of application. Additionally, other new drugs should be also analyzed for their effect on CNS recurrence.

Lenalidomide also exhibits varying effects in different DLBCL subtypes. Research revealed that patients having non‐GCB/ABC DLBCL had higher response to lenalidomide versus the GCB/ABC subtype, predominantly due to the overactivation of NF‐kB and the higher level of interferon regulatory factor‐4 (IRF4).^27^, ^37^, ^38^, ^39^, ^40^ In the REMARC trial, researchers observed that the PR‐to‐CR conversion in cohorts using lenalidomide and placebo was similar.^21^ A speculation was thus proposed that the clinical effect of lenalidomide maintenance is dependent on a kind of immunomodulatory mechanism rather than the direct tumoricidal effect generated by the interferon‐stimulated gene upregulation, and this effect is independent of cell of origin (COO). This speculation could be supported by other two studies on lenalidomide maintenance treatment.^41^, ^42^ Here, no remarkable difference in PFS of non‐GCB and GCB subtypes receiving lenalidomide maintenance treatment (*p* = 0.49) was indicated, which also supports the above speculation. The specific mechanism of action of lenalidomide could help formulate more targeted, individualized treatment for patients with aggressive DLBCL.

Hematological toxicity (such as neutropenia) and non‐hematological toxicity (such as cutaneous reaction) are common adverse reactions during treatment, while the majority of them is controllable under supportive treatment. In this study, no severe complication or treatment‐related death was observed. Five patients (10%) received lenalidomide with a dose reduction and completed 2 years of maintenance therapy. In all, lenalidomide is safe when applied in the maintenance therapy after R‐CHOP induction treatment, and it is much more suitable in the elderly and frail patients. Overall, this study reflects the real‐world efficacy of lenalidomide and provides a foundation for clinical translation. However, the number of patients in our study is small and it is just a single center retrospective trial.

In conclusion, this study identifies lenalidomide as an effective maintenance therapy in responding DLBCL patients with R‐CHOP induction treatment, with controllable toxicity.

## AUTHOR CONTRIBUTIONS

**Xiaoyan Wang:** Conceptualization (equal); data curation (lead); formal analysis (equal); investigation (lead); methodology (equal); visualization (lead); writing – original draft (lead); writing – review and editing (equal). **Lu Yu:** Data curation (supporting); formal analysis (supporting); investigation (supporting); visualization (supporting). **Xinlu Jiang:** Data curation (supporting); formal analysis (supporting); investigation (supporting); visualization (supporting). **Kaiyang Ding:** Conceptualization (lead); formal analysis (lead); funding acquisition (lead); methodology (lead); project administration (lead); resources (lead); supervision (lead); writing – original draft (equal); writing – review and editing (lead).

## FUNDING INFORMATION

This work was supported in part by the Key Research and Development Project of Anhui Province [grant number 1804 h08020249].

## CONFLICT OF INTEREST STATEMENT

The authors declare that they have no competing interests.

## ETHICAL APPROVAL STATEMENT

The Ethics Committee of the Anhui Provincial Hospital Affiliated to Anhui Medical University granted permission for the investigation.

## PATIENT CONSENT TO PARTICIPATE

All patients signed an informed consent before treatment.

## Data Availability

Data Availability statement: the data that support the findings of this study are available from the corresponding author upon reasonable request
